# A clinical study of thoracoscopy-assisted mitral valve replacement concomitant with tricuspid valvuloplasty, with domestically manufactured pipeline products for cardiopulmonary bypass

**DOI:** 10.1186/s13019-014-0160-2

**Published:** 2014-10-02

**Authors:** Hua Cao, Qiang Chen, Qian-Zhen Li, Liang-Wan Chen, Gui-Can Zhang, Dao-Zhong Chen, Zhi-Huang Qiu, Yun-Nan Hu, Jia-Jun He

**Affiliations:** Department of Cardiovascular Surgery, Union Hospital, Fujian Medical University, Xinquan Road 29#, Fuzhou, 350001 P. R China

**Keywords:** Thoracoscopy assistance, Minimally invasive, Mitral valve surgery

## Abstract

**Objective:**

To discuss the feasibility and experience of treating valvular heart diseases with thoracoscopy-assisted mitral valve replacement concomitant with tricuspid valvuloplasty, with domestically manufactured pipeline products for cardiopulmonary bypass.

**Methods:**

A total of 135 patients with valvular heart disease were admitted to our hospital between January 2011 and January 2013. They received thoracoscopy-assisted mitral valve replacement concomitant with tricuspid valvuloplasty, with domestically manufactured pipeline products. A cardiopulmonary bypass with domestically-manufactured pipeline products was established during the surgery. The procedure was accomplished with the assistance of thoracoscopy through a small incision in the right chest wall.

**Results:**

All 135 patients underwent a successful surgery, and were followed up for the duration of half a year to two years. None of them displayed any evidence of complications. Our procedure had the advantage of fewer complications and a significantly shortened time period for the patient care and hospitalization. As opposed to imported pipeline products for cardiopulmonary bypass, our procedure had the advantage of similar clinical results at a lower cost.

**Conclusions:**

Thoracoscopy-assisted mitral valve replacement concomitant with tricuspid valvuloplasty was proved to be a safe and effective method for cardiopulmonary bypass, with the use of domestically manufactured pipeline products.

**Electronic supplementary material:**

The online version of this article (doi:10.1186/s13019-014-0160-2) contains supplementary material, which is available to authorized users.

## Background

Valvular heart disease is a commonly acquired heart disease in adults. The traditional treatment method has been the open-heart surgery under cardiopulmonary bypass. In recent years, more clinicians and patients favored the application of thoracoscopy technology in the heart surgery due to its multiple advantages. These included less surgical trauma, shorter hospital stay, an almost intact chest wall after the procedure without any steel wires left, and good cosmetic effects [1]-[4]. We performed thoracoscopy-assisted mitral valve replacement concomitant with tricuspid valvuloplasty with the use of the domestically-manufactured pipeline products for the cardiopulmonary bypass on 135 cases of valvular heart diseases between January 2011 and January 2013. The results were satisfactory and are reported below.

## Methods

The present study was approved by the ethics committee of Union Hospital, Fujian Medical University and adhered to the tenets of the Declaration of Helsinki. Additionally, the written informed consent was obtained from the patients.

### Clinical data

This study contained 135 patients, including 60 males and 75 females. Their ages ranged between 25 and 62 years old (42.2 ± 11.6 years old). Of those, 78 cases had rheumatic heart disease, mitral stenosis combined with tricuspid insufficiency, 42 patients were diagnosed with rheumatic heart disease, mitral stenosis with regurgitation combined with tricuspid insufficiency, and 15 cases had mitral and tricuspid insufficiency. All the patients were clearly diagnosed using echocardiography. Those with other cardiac malformations and aortic valve diseases were excluded. Ninety patients had a mild to moderate pulmonary hypertension. Any cases with pre-operative pulmonary infection were treated using standard antibiotics, and those with pre-operative chronic heart failure were treated with oral cardiotonic agents and diuretics.

### The pipeline products

**Figure 1:**
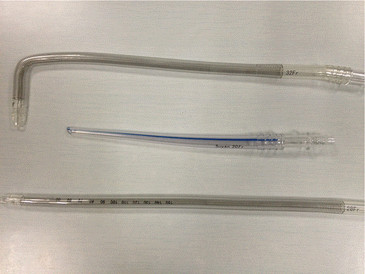
**20** ***f*** **-single-lumen arterial** **cannula** **, 28** ***f*** **-single-lumen venous** **cannula** **and 32** ***f*** **-right-angled superior vena cava** **cannula.**

The pipeline products for cardiopulmonary bypass used in this study were all from Tianjin Plastics Research Institute Co., Ltd, China. 20-24 *f*-single-lumen arterial intubations were used for the femoral artery, 28-30 *f*-single-lumen venous intubations were used for the femoral vein, and 32-34 *f*-right-angled superior vena cava cannulas were used for the superior vena cava (Figure 1).

### Surgical procedures

All the patients were under single-lumen endotracheal intubation and intravenous-inhalation combined anesthesia. They were placed in the supine position with the right chest elevated by 45°. The right upper arm was raised and stabilized beside the head. A 3-cm long longitudinal incision was made along the right groin. The femoral artery and vein were separated and a purse was sewn in each. A 4-cm long anterolateral incision was made in the right anterior chest. The chest was entered through the fourth intercostal space, which functioned as the surgical entrance (incision 1). A 1-cm long incision was made along the mid-axillary line. From there, the chest was entered through the fourth intercostal space, which functioned as the entrance of thoracoscopy and left atrial suction tube (incision 2). Another 1-cm long incision was made along the anterior axillary line and the chest was entered through the third intercostal space. This functioned as the entrance of the superior vena cava drainage tube, occlusion band, and ascending aortic occlusion clamp (incision 3). Through incision 1, a pericardial incision was made above the right phrenic nerve. The incision went up to the ascending aortic pericardium reflection, and then down to the root of the inferior vena cava. The lower edge of the pericardium was intermittently stitched with five to seven sutures of traction thread, which were elicited through incision 1 and 2, and exposed the right atrium and root of the superior vena cava. After systemic heparinization, the femoral artery and vein were intubated. The flow in the femoral artery and vein was bypassed and the tidal volume was halved, which led to a partial collapse of the right lung. A purse was then sutured at the root of the superior vena cava, and a right-angled vena cava cannula was inserted. Following a full flow bypass, the superior and inferior vena cava was interrupted by a sleeve-banding method and the ventilator was stopped, leading to a complete collapse of the right lung. A double needle purse with gasket was sutured at the aortic root, and a perfusion needle filled with cardiac protective solution was inserted into it. At the entrance to the right superior pulmonary vein, a purse was sutured and a left atrial drainage tube was inserted into that. At the aortic root through incision 2, an aortic occlusion clamp was inserted into that so as to block the ascending aorta [5]. An antegrade perfusion of the cold-blooded cardioplegia was performed and the heart surface was covered with the ice-cold saline to cool and protect the myocardia.

Once the cardiac arrest was satisfactorily achieved, an incision parallel to the atrioventricular groove was made so as to open the right atrium and interatrial septum. Three traction threads were sutured in the interatrial septum, which were used to expose the left atrium and mitral valve by traction. The left atrial thrombus was explored and cleared, the diseased valves were removed, and the new prosthetic valves were implanted using an intermittently suturing method. The incision in the interatrial septum was continuously sutured. Furthermore, before the closure of the interatrial septum, the lung was inflated in order to fill the left atrium with blood. A BalMedic "C"-shaped soft ring was accordingly implanted in the tricuspid annulus. The incision in the right atrium was then closed and the lung was inflated to fill the right atrium with blood.

Air was sucked and exhausted through the perfusion needle at the root of the ascending aorta. The temperature was then increased and the ascending aorta was opened, leading to a cardiac resuscitation. At the same time, circulation was restarted and the cardiopulmonary bypass was gradually stopped. All the tubes were removed, the wound bleeding stopped, and the pericardial incision was intermittently sutured. A thoracic closed drainage tube was placed at the site of the incision 2. After the surgery, the patients were sent to the Intensive Care Unit (ICU), and endotracheal intubation was removed when they had regained consciousness. The patients also received a standard anti-coagulant therapy.

### Statistical analysis

Continuous data were presented as mean ± standard deviation and range. Clinical parameters between the two groups were compared with the independent samples t-test. A p value of <0.05 was defined as statistical significance. In order to support our point of view, we had compared our results with previous clinical unpublished record which contained conventional open-heart surgery and thoracoscopy assistance and imported pipeline products for cardiopulmonary bypass.

## Results

All the cases underwent a successful surgery and were alive. The operation took between 3.5 to 5.6 hours (4.6 ± 1.1). The cardiopulmonary bypass lasted between 85-146 minutes (115.5 ± 27.3) and the ascending aorta was blocked for 44-73 minutes (60.2 ± 8.1). The post-operative thoracic closed drainage volume was between 20-400 ml (100.6 ± 35.2). The post-operative ICU stay lasted for 6-12 hours (7.5 ± 1.0) and the post-operative hospitalization took another 7-20 days (9.8 ± 2.6).

There were some post-operative complications, including: five cases of right pneumothorax and 15 patients with right pleural effusion with a need for catheters for drainage; five cases with post-operative lung infection treated with antibiotics; two patients with fat liquefaction at the inguinal incision, which was healed after a dressing change and a secondary suture; twenty cases of peri-operative arrhythmias, including supraventricular tachycardia, atrial flutter, and atrial fibrillation, which went back to normal shortly after the treatment; and two patients with peri-operative ventricular fibrillation, whom successfully recovered using cardiopulmonary resuscitation.

All the patients were post-operatively followed up for a period of half a year to two years using echocardiography and electrocardiogram examination. The results showed that the prosthetic valve was normally positioned and functioned well. Furthermore, the tricuspid valve function significantly improved as compared to its pre-operative condition.

Table 1 shows the comparison between this group of patients and another group of 125 patients who had a previous conventional open-heart surgery in our division. (unpublished data) There was no significant difference in patient choices between these two groups. The results showed that aortic blocking and cardiopulmonary bypass took a longer time in the thoracoscopic group as compared to the conventional group (*P* < 0.05). Also, there was no noteworthy difference in the post-operative ventilator assistance time between these two groups (*P* > 0.05). The post-operative 24-hour pleural fluid volume, post-operative ICU supervision time, and post-operative hospitalization time were greatly reduced in the thoracoscopic group as compared to those of the conventional group (*P* < 0.05).

**Table 1:** **Data comparison between this group of patients and another group of 125 patients (unpublished) having conventional open heart surgery in our division**

Item	Thoracoscopic group	Conventional group	***P***
**Sex(M:F)**	60:75	54:71	>0.05
**Age(months)**	42.2 ± 11.6	43.1 ± 10.8	>0.05
**Weight(kg)**	45.3 ± 10.5	48.2 ± 11.1	>0.05
**Cardiopulmonary bypassing time(min)**	115.5 ± 27.3	65.5 ± 5.6	<0.05
**Aortic occlusion clamping time(min)**	60.2 ± 8.1	40.3 ± 7.8	<0.05
**Ventilator assistance time(h)**	6.5 ± 1.5	6.3 ± 1.3	>0.05
**Postoperative 24 hour pleural fluid volume(ml)**	100.6 ± 35.2	255.8 ± 64.8	<0.05
**Postoperative ICU supervision time(h)**	8.5 ± 3.0	13.3 ± 4.2	<0.05
**Postoperative hospitalization time(d)**	9.8 ± 2.6	12.5 ± 3.8	<0.05

Table 2 shows the comparison between this group of patients and a group of 87 other cases in our division who were previously operated using thoracoscopy assistance and imported pipeline products for cardiopulmonary bypass. (unpublished data) There was no significant difference in the patient choices between these two groups. The results showed that there was a less notable difference in the aortic blocking time, cardiopulmonary bypass time, post-operative ventilator assistance time, post-operative 24-hour pleural fluid volume, post-operative ICU supervision time, and post-operative hospitalization time (*P* > 0.05). The cost, however, was significantly higher for the group using imported pipeline products for cardiopulmonary bypass as compared to that of the conventional group using domestically manufactured ones (*P* < 0.05).

**Table 2:** **Data comparison between this group of patients and another group of 87 patients (unpublished) using thoracoscopy assistance and imported pipeline products for cardiopulmonary bypass in our division**

Item	Domestic product group	Imported product group	***P***
**Sex (M:F)**	60:75	38:49	>0.05
**Age (months)**	42.2 ± 11.6	45.3 ± 9.9	>0.05
**Weight (kg)**	45.3 ± 10.5	47.5 ± 12.3	>0.05
**Cardiopulmonary bypassing time (min)**	115.5 ± 27.3	108.3 ± 22.5	>0.05
**Aortic occlusion clamping time (min)**	60.2 ± 8.1	59.4 ± 10.2	>0.05
**Ventilator assistance time (h)**	6.5 ± 1.5	6.9 ± 1.2	>0.05
**Postoperative 24 hour pleural fluid fluid volume(ml)**	100.6 ± 35.2	95.6 ± 42.1	>0.05
**Postoperative ICU supervision time(h)**	8.5 ± 3.0	8.2 ± 2.8	>0.05
**Postoperative hospitalization time(d)**	9.8 ± 2.6	9.5 ± 2.8	>0.05
**Hospitalization cost(RMB)**	60539.5 ± 1341.2	69806.7 ± 998.5	<0.05

## Discussion

Valvular heart disease is a commonly acquired heart disease, especially in the developing countries. Valve replacement surgery for rheumatic heart disease is a clinically common procedure in the cardiac surgery division. Conventional open-heart surgery has the advantage of fully exposing each chamber of the heart and large vessels, so that all kinds of emergency situations in the surgery could be easily handled. Unfortunately, to accomplish this procedure, it needs the sternum sawed which increases the risk of wound pain, infection or delayed healing. After the use of the methods like an incision in the center of the chest, a small parasternal incision, and an incision from the partial sternotomy, thoracoscopy technology has been gradually increased in the cardiovascular surgery. In 1996, Carpentier et al. completed the first mitral valvuloplasty using a video-assisted thoracoscopy. This was a start to the era of the thoracoscopy-assisted valve surgery [6]. Compared to the conventional cardiac surgery, thoracoscopy-assisted heart valve replacement has many advantages, including: a smaller incision, less trauma, less blood loss during the surgery, less post-operative pain, quicker recovery, and shorter hospital stay [7]-[9].

However, the heart and large vessels are not well exposed in the thoracoscopy-assisted heart surgery. Compared to the conventional open heart surgery, the procedure of establishing a cardiopulmonary bypass in the thoracoscopy-assisted heart surgery is difficult, and normally a periphery cardiopulmonary bypass is used. The right femoral artery is mostly chosen for the arterial perfusion. Pre-operative ultrasound examination should be performed to exclude the presence of a severe aortic sclerosis and dissecting aortic aneurysm to help avoiding a cerebral embolism and other severe complications.

The main feature of this group of patients was the 20-24 *f*-cannula that was used in the femoral artery intubation (Tianjin Plastics Research Institute Co., Ltd). In the flow bypass arterial perfusion resistance might be significantly increased due to an arterial spasm. During this part of the process, a timely communication should be maintained with the surgeon. Furthermore, both depth and direction of the intubation should be accordingly adjusted. A 28-30 *f*-single stage pipeline (Tianjin Plastics Research Institute Co., Ltd) was used for the femoral vein, and the drainage port was placed inside the inferior vena cava to ensure a full venous drainage during the bypass process. A 32-34 *f*-right-angled superior vena cava cannula (Tianjin Plastics Research Institute Co., Ltd) was used for the superior vena cava. In order to prevent the safety and perfusion volume of the cardiopulmonary bypass from being affected due to the high arterial resistance, we chose a larger cannula for the femoral artery and vein. Besides, lung ventilation can only be stopped by the anesthesia which was performed after the occlusion of the superior and inferior vena cava, otherwise heart and brain hypoxia could have happened since the retrogradely perfused arterial blood in the femoral artery could not reach the aortic root, so the anesthetist should closely communicate with the surgeon and cardiopulmonary bypass perfusionist during the surgery.

The imported pipeline products commonly used in the clinic have been manufactured by Edwards Lifesciences Corporation and cost 4093.74 RMB [10]. However, the pipeline products used in the current group of patients were manufactured by Tianjin Plastics Research Institute Co., Ltd and only cost 179.28 RMB. As shown in Table 2, these two types of pipeline products had similar clinical effects, but the cost of the one used for the current group of patients was much lower. This could be especially important for the low income countries that want to popularize the thoracoscopy technology. For some patients with thin femoral arteries and veins, we still chose to use the imported pipeline products.

Improvements on the cardiopulmonary bypass technology have caused a decrease in the use of pipes in the surgical field. Thoracoscopy technology allows the surgical field to be clearly exposed. These two processes enable a small incision mitral valve replacement surgery to be better implemented. The lung doesn't need to be collapsed, so only a simple single-lumen endotracheal intubation is required, which avoids the use of a more expensive double-lumen endotracheal intubation. The establishment of a femoral artery-femoral vein bypass and subsequent reduction of tidal volume not only guarantees enough oxygen supply, but also exposes the root of the superior vena cava. The use of the domestically-produced right angled superior vena cava cannula and the exit of the chest through incision 3, has no effect on the surgical field and avoids the use of a more expensive imported bipolar femoral vein cannula.

We made an anterolateral small incision in the right fourth intercostal space that could be distracted to a sufficient size during the surgery without a need to transversely break the ribs. In this way, the operation could be directly performed through that small incision. The incision was about 4 cm long, which was the required length for implanting a prosthetic valve into the chest as well as a combined direct vision for surgery. The incision should be as minimally invasive and good-looking as possible [11],[12]. Since the distraction space for the parasternal incision is small and the length for the incision extension is limited, in the occasion of any difficulty during the operation, a rib needs to be broken.

During the surgery, particular attention was made to cuffing the superior and inferior vena cava and making sure to follow along the anatomical space not to damage the vena cava. The damage to the back wall of the vena cava could be difficult to repair under thoracoscopy, so the incision should be decisively expanded if necessary. The aortic purse stitches should be as shallow as possible with a positive stitching, and the needle should follow along the curvature. For the patients with a severe membrane calcification, this surgery is not recommended due to its high surgical risk and difficulty in exposing the subvalvular. When the left atrium has a thrombus inside, ligation of the root of the left atrial appendage is necessary during the surgery. Closing the left atrial appendage could be done in the left atrium; however, this could be very risky and upon the left atrium bleeding, the consequences could be disastrous. Lastly, right before the completion of the surgery, any bleeding should be stopped. Most importantly, it should be checked whether the intercostal vessels are bleeding.

Video thoracoscopy-assisted cardiac surgery is different from the traditional surgery, since it introduces changes in the vision field and the operating habits of the surgeons. A given operation is finished with deep instrumentation practices. Practices like peeling, cutting, stitching, and bleeding stoppage are performed differently in deep surgical locations as compared to when direct vision practices are utilized [13]. This is mainly due to the difference in depth distance, vision, orientation, and motor coordination. Cardiopulmonary bypass and aortic blocking took a longer time in this group of patients, indicating that thoracoscopy-assisted open-heart surgery could be difficult. Therefore, the implementation should be strictly in accord with the surgical indications in order to achieve the same effect and safety as that of the traditional surgery. Based on our observation, the indicators for the thoracoscopy-assisted open-heart surgery included: an age of less than 60 years, absence of any pleural and pericardial adhesions, pure mitral valve disease or mitral combined with tricuspid valve disease, a left atrial diameter of greater than 45 mm, a cardiac function below grade III, and the absence of any lesion or malformation in the nearby large vessels. When issues such as poor visibility, operational difficulty, unforeseen circumstances, or a combination with other lesions need to be addressed during the surgery, a small incision could be expanded up or down and the surgery could be switched into a direct-vision operation. Of course, selecting the cases is always relative. With the advancement of technology, the thoracoscopic technique is increasingly being used in the high-risk cases [14]-[16].

Thoracoscopy can provide surgeons with a clear amplified two-dimensional surgical field. The entire right pleural cavity and cardiac anastomosis can be clearly observed, which is conducive to the post-operative bleeding stoppage. In this group of patients, the post-operative pleural fluid volume was less than that of the conventional surgery group. Few incisions, light pain response, and an intact sternum could be beneficial to the recovery of patients' post-operative complications and coughing functions. As a result, the ICU and hospital stay-time was less than that of the traditional group in this group of patients, which was consistent with previous reports [17],[18]. Therefore, this procedure could be much easier to be accepted by patients, especially when clinical costs could be relatively reduced. More importantly, it could be easier to be popularized in the low-income countries [19],[20].

This study had many inadequacies. Only a small number of patients were included in this study and they were followed up for a short period of time, which caused a limited experience. A larger number of cases and longer follow-up times would be needed for future studies. This current procedure was not intended to replace the traditional one. In fact, many patients had a variety of surgical options and the decision on which specific surgical approach should have been chosen was combined with the actual situations, such as each patient's condition and needs, severity of the disease, the doctor's experience, the hospital's equipment, and economic situations [21].

## Conclusions

In summary, we believe that it is safe and effective to treat valvular heart diseases with the thoracoscopy-assisted mitral valve replacement concomitant with tricuspid valvuloplasty with domestically manufactured pipeline products for cardiopulmonary bypass. It is minimally invasive and at the same time has cosmetic effects. Domestically-manufactured pipeline products for cardiopulmonary bypass can minimize the economic pressures on the patients and the society, and achieves similar surgical results as compared to the imported pipeline products for the cardiopulmonary bypass. Therefore, we conclude that it could be worthy recommending thoracoscopy assisted mitral valve replacement concomitant with tricuspid valvuloplasty with domestically manufactured pipeline products for cardiopulmonary bypass to treat valvular heart diseases.

## Authors’ original submitted files for images

Below are the links to the authors’ original submitted files for images. Authors’ original file for figure 1
