# Supplementary figures and images for: A clinical study of thoracoscopy-assisted mitral valve replacement concomitant with tricuspid valvuloplasty, with domestically manufactured pipeline products for cardiopulmonary bypass
Source: J Cardiothorac Surg. 2014 Oct 2;9:160. doi: 10.1186/s13019-014-0160-2 (PMC4192743; doi:10.1186/s13019-014-0160-2)

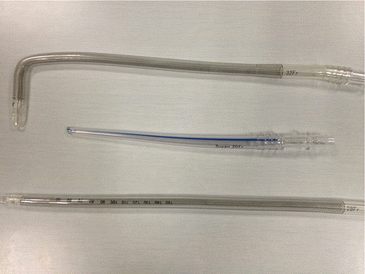

Supplement: Supplementary file 1 — Authors’ original file for figure 1 [file 13019_2014_160_MOESM1_ESM.gif]
